# Sex-Specific Abnormalities and Treatment-Related Plasticity of Subgenual Anterior Cingulate Cortex Functional Connectivity in Chronic Pain

**DOI:** 10.3389/fpain.2021.673538

**Published:** 2021-07-12

**Authors:** Natalie R. Osborne, Dimitri J. Anastakis, Junseok Andrew Kim, Rima El-Sayed, Joshua C. Cheng, Anton Rogachov, Kasey S. Hemington, Rachael L. Bosma, Camille Fauchon, Karen D. Davis

**Affiliations:** ^1^Krembil Research Institute, Krembil Brain Institute, University Health Network, Toronto, ON, Canada; ^2^Institute of Medical Science, University of Toronto, Toronto, ON, Canada; ^3^Toronto Western Hospital, University Health Network, Toronto, ON, Canada; ^4^Department of Surgery, University of Toronto, Toronto, ON, Canada

**Keywords:** neuropathic pain, subgenual anterior cingulate cortex, functional connectivty, carpel tunnel syndrome, treatment, pre-frontal cortex, fMRI, sex differences

## Abstract

The subgenual anterior cingulate cortex (sgACC) is a key node of the descending antinociceptive system with sex differences in its functional connectivity (FC). We previously reported that, in a male-prevalent chronic pain condition, sgACC FC is abnormal in women but not in men. This raises the possibility that, within a sex, sgACC FC may be either protective or represent a vulnerability to develop a sex-dominant chronic pain condition. The aim of this study was to characterize sgACC FC in a female-dominant chronic pain condition, carpal tunnel syndrome (CTS), to investigate whether sgACC abnormalities are a common feature in women with chronic pain or unique to individuals with pain conditions that are more prevalent in the opposite sex. We used fMRI to determine the resting state FC of the sgACC in healthy controls (HCs, *n* = 25, 18 women; 7 men) and people with CTS before (*n* = 25, 18 women; 7 men) and after (*n* = 17, 13 women; 4 men) successful surgical treatment. We found reduced sgACC FC with the medial pre-frontal cortex (mPFC) and temporal lobe in CTS compared with HCs. The group-level sgACC-mPFC FC abnormality was driven by men with CTS, while women with CTS did not have sgACC FC abnormalities compared with healthy women. We also found that age and sex influenced sgACC FC in both CTS and HCs, with women showing greater FC with bilateral frontal poles and men showing greater FC with the parietal operculum. After surgery, there was reduced sgACC FC with the orbitofrontal cortex, striatum, and premotor areas and increased FC with the posterior insula and precuneus compared with pre-op scans. Abnormally reduced sgACC-mPFC FC in men but not women with a female-prevalent chronic pain condition suggests pain-related sgACC abnormalities may not be specific to women but rather to individuals who develop chronic pain conditions that are more dominant in the opposite sex. Our data suggest the sgACC plays a role in chronic pain in a sex-specific manner, and its communication with other regions of the dynamic pain connectome undergoes plasticity following pain-relieving treatment, supporting it as a potential therapeutic target for neuromodulation in chronic pain.

## Introduction

The subgenual anterior cingulate cortex (sgACC) plays an important role in the descending pain modulation (DPM) pathway. It is associated with endogenous opioidergic pain inhibition (1, 2) and is structurally and functionally connected to key nodes within the DPM pathway, including the periaqueductal gray (PAG), rostroventral medulla (RVM), and medial pre-frontal cortex (mPFC) (3–6). In healthy individuals, stronger sgACC functional connectivity (FC) with the DPM is associated with reduced temporal summation of pain (7), and habituation to sustained painful stimuli corresponds to sgACC activity (8). Importantly, there are sex differences in sgACC FC in healthy individuals. An fMRI study of static resting state (rs) sgACC FC in our lab found that women had greater connectivity with nodes of the DPM pathway (PAG and raphe nuclei) while men had greater connectivity with the salience network (3). Furthermore, our recent magnetoencephalography (MEG) study of healthy adults found sex differences in dynamic FC in the sgACC across multiple frequency bands (9).

Dysfunction in the DPM system (10–12), including the rostral ACC (13), is a common finding in chronic pain, but the extent and the character of these abnormalities differ between women and men (14). Sex differences in sgACC effective connectivity may underlie sex differences in brain responses to painful stimuli as well as symptoms in chronic pain (15). Both fMRI and MEG studies in our lab have found abnormalities, specifically in the sgACC, across chronic pain conditions. A MEG study of patients with multiple sclerosis identified abnormal static and dynamic FC that was particularly pronounced in patients with neuropathic pain (16). In another study, a graph-theory analysis of rsFC in patients with low-back pain due to ankylosing spondylitis (AS) found atypical modular segregation in the mid cingulate cortex and sgACC in women with chronic pain, with stronger interactions between nodes within the sgACC module and weaker interactions with nodes outside of it (17). A FC study that focused on the sgACC in a subset of these chronic pain patients found abnormal sgACC FC only in the women. Compared with healthy women, women with AS had increased FC with nodes of the default mode and salience networks but decreased FC with the left inferior frontal gyrus and dorsolateral pre-frontal cortex (DLPFC)(18).

Interestingly, AS is one of the few chronic pain conditions that are more prevalent in men than women (19). There are numerous sex differences in pain physiology and gendered cognitive and social influences on pain that could translate to differences in chronic pain prevalence and presentation (20). Exactly why a given chronic pain condition is more dominant in a particular sex, and why individuals develop a condition that is significantly less prevalent in their sex is not clear. In the present study, we aimed to explore if sex-specific abnormalities in sgACC FC exist in people with a chronic pain condition that is ~3 times more prevalent in women than men, carpal tunnel syndrome (CTS) (21, 22). CTS is the most common entrapment neuropathy, characterized by pain, numbness, and tingling in the hands caused by chronic compression and subsequent damage to the median nerve in the wrist (23, 24). Surgical release of the carpal tunnel leads to successful resolution of symptoms in approximately 80% of CTS patients within 3 months (25), making it a good condition to study treatment-related brain plasticity (26).

Treatment-related plasticity in the sgACC has not been well-studied in chronic pain, although this region has been a target to alleviate major depressive disorder, using deep brain stimulation (DBS) (27) and non-invasive brain stimulation. Interestingly, FC of the sgACC appears to predict responses to cognitive behavioral therapy for depression (28), response to medication (29) and non-invasive brain stimulation treatments (30–32), and is used to guide stimulation site placement. Furthermore, posttreatment sgACC FC with the default mode network, DLPFC, and insula was associated with symptom reduction after transcranial magnetic stimulation (31), and a small study using intracranial recordings from DBS electrodes placed in the sgACC showed changes in beta oscillations, corresponding to normalized responses to an emotional task, following 6 months of DBS treatment (33).

Despite a growing understanding of the role of the sgACC as a target for treating depression, its potential as a future therapeutic target for pain treatments has not been fully realized. Thus, in this study, our aim was to characterize sgACC FC in individuals with chronic pain and in those whose pain has resolved following treatment to better understand this region's role in chronic pain in women and men. In this longitudinal study, we collected rs fMRI in 25 healthy controls (HCs) and 25 CTS patients before CTS release surgery, 17 of whom returned for a post-op fMRI. We hypothesized that sgACC FC would be abnormal in individuals with CTS, and this pattern of abnormalities would be influenced by sex. We also hypothesized that sgACC to whole brain FC patterns would differ within individuals when experiencing ongoing chronic pain (i.e., pre-op CTS) compared with a pain-free state (i.e., post-op CTS).

## Materials and Methods

### Participants

This study included 25 (18 women, 7 men; mean age ± SD 49.6 ± 12 years) patients with chronic pain associated with CTS and 25 sex-matched healthy controls (HCs, mean age ± SD 45.7 ± 9.4 years). CTS patients were recruited from the clinic of Dr. Dimitri Anastakis, a hand surgeon at the Toronto Western Hospital Hand Program. The HCs were recruited with advertisements posted throughout hospitals of the University Health Network in Toronto, Ontario, Canada. All the participants provided informed, written consent to the study procedures that were approved by the University Health Network Research Ethics Board.

A diagnosis of CTS was confirmed through clinical examination by Dr. Anastakis and his team. Each clinical assessment involved a thorough history of symptomology and clinical tests, including Tinel's Sign, Phalen's Test (34), Pressure Provocation Test (35), and two-point discrimination tests as well as assessments of grip strength, key pinch, muscle atrophy, and abductor pollicis brevis tone (22). Nerve conduction studies were done in cases with diagnostic uncertainty, but electrodiagnostic tests were not required for inclusion in the study because CTS is a clinical diagnosis (36). Our inclusion criteria included patients between the ages of 18 to 70 years of age with unilateral or bilateral CTS who failed conservative management (defined as three consecutive months of nightly splint wearing) and were recommended for carpal tunnel release surgery. Our exclusion criteria were (1) polyneuropathy/other peripheral neuropathy (e.g., double crush syndrome, cervical radiculopathy, and ulnar neuropathy), (2) atypical innervation of the hand (e.g., Martin-Gruber or Riche-Cannieu anastomoses), (3) previous carpal tunnel release surgery, (4) rheumatoid arthritis or degenerative osteoarthritis in the hand, (5) diabetes mellitus, renal disease or metabolic disorder, (6) neurodegenerative disorders (e.g., dementia, Parkinson's disease), (7) other chronic pain conditions, (8) psychiatric disorders (e.g., personality and eating disorders); however, patients with anxiety or depression were not excluded, and (9) pregnancy, claustrophobia, and contraindications for MRI. HCs were included if they were between the ages of 18 and 70 and excluded if they had any of the nine criteria above, as well as (1) current acute pain or diagnosis of a chronic pain condition and (2) diagnosis of any psychiatric or neurological disorder, including depression and anxiety.

### Study Protocol

The study protocol for CTS patients involved both a presurgical assessment prior to surgical release of one or both of their carpal tunnels, and a post-surgical assessment for a subset of patients. At each session, neuroimaging data were acquired and questionnaires were collected. All the surgeries were performed by Dr. Anastakis. Of the patients that enrolled in the study, 17 returned for the postsurgical follow-up assessment. Post-op visits were scheduled for at least 3 months after surgery to allow sufficient time for the median nerve to heal and regenerate. In the cases where a patient had significant bilateral CTS and elected to have surgery on both hands, the post-op visit was collected at least 3 months after the second surgery. The HCs were assessed in a single visit, which included neuroimaging data acquisition and questionnaires.

### Questionnaires

The severity of CTS was assessed with the Boston Carpal Tunnel Questionnaire (BCTQ), a validated tool (37, 38) that contains a subscale for symptoms and another subscale to assess function. The symptom severity scale consists of 11 questions about the occurrence and duration of sensations such as pain, numbness, and tingling. They are rated 1–5, with 1 representing a “normal” response (no symptoms) and 5 indicating “very serious” or constant symptoms. The functional status scale contains eight questions that assess how CTS affects hand function, such as writing or opening jars, and each question is rated from 0 (No difficulty) to 5 (Cannot perform the activity at all due to hands and wrists symptoms). Scores for both subscales are calculated by tallying the numerical answers and dividing the total by the number of questions for that subscale. The resulting score for each subscale is then added to get the total BCTQ score. Two represents a “normal” BCTQ score, while the most severe score is 10. The patients who had bilateral CTS completed a BCTQ questionnaire for each hand, and, here, we report the BCTQ score for the most symptomatic hand.

Two questionnaires were used to assess pain in the patients. The pain DETECT questionnaire (39) was used to assess the likelihood of the presence of neuropathic pain. The pain DETECT scores can range from 0 to 38, with scores 12 and under indicative of pain that is unlikely to be neuropathic and thus categorized as “non-neuropathic.” Scores between 13 and 18 indicate a “mixed” pain etiology in which a neuropathic component is likely present, and scores 19 and above indicate high likelihood of neuropathic pain. The Brief Pain Inventory (40) (BPI) was used to assess pain severity at present, average pain, and pain at its worst and least in the past 24 h (41). The BPI uses scales from 0 (no pain) to 10 (pain as bad as you can imagine). Here, we report the average of these four ratings as the patient's “average pain severity.” The participants self-identified their sex in a sociodemographic questionnaire with the binary options of male and female. All the study participants completed the Beck Depression Inventory (BDI), a 21-question measure of cognitive and affective symptoms of depression. Each question is scored from 0 to 3, with a total score range of 0–63 (42). Depression scores are classified as none to minimal (0–13), mild (14–19), moderate (20–28), and severe (29–63).

### Neuroimaging Acquisition

Neuroimaging data were collected with a 3T GE Signa MRI scanner fitted with an 8-channel phase-array head coil (GE Medical Systems, Chicago, IL). The neuroimaging acquisition protocol included a high resolution T1-weighted anatomical scan (3D IR-FSPGR sequence; 180 axial slices; repetition time (TR), 7.8 ms; echo time (TE), 3 ms; flip angle, 15°; 256 × 256 matrix; 1 × 1 × 1 mm^3^ voxels) and a T2^*^-weighted eyes-closed resting-state fMRI scan, lasting 9 min and 14 s (echo-planar imaging sequence; 36 slices; TR, 2,000 ms; TE, 30 ms; 64 × 64 matrix; 3.125 × 3.125 × 3.125 mm^3^ voxels).

### Pre-processing of Functional MRI Data

For data preprocessing, we used the fMRI Expert Analysis Tool (FEAT) in FMRIB Software Library (FSL) version 5.0.1. The first four volumes of the resting-state scan were removed. We used FEAT's Brain Extraction Tool (BET) to remove non-brain voxels from the functional data. Motion correction was performed using Motion Correction FMRIB's Linear Image Registration Tool (MCFLIRT). The participants' absolute and relative root mean square displacement (RMS) was inspected to ensure values did not exceed the cutoff of 2.5 mm (absolute RMS) and 0.30 mm (relative RMS). T1-weighted anatomical images were skull stripped with OptiBET (43). FMRIB's Linear Image Registration Tool (FLIRT)(44) (rigid body transformation with six degrees of freedom) was used to register each participant's functional images to his or her skull-stripped T1-weighted anatomical image, followed by non-linear registration to MNI152-2 mm space with FMRIB's Non-linear Image Registration Tool (FNIRT). To further remove physiological and scanner-related noise, we used aCompCor (45), as described previously (46). This process regresses out the top five white matter and cerebrospinal fluid components, and six motion parameters out of the functional data. Finally, spatial smoothing was applied, using a 4-mm FWHM Gaussian spatial smoothing kernel, and temporal filtering was performed, using a high-pass filter (0.01 Hz cutoff) to remove scanner-related signal drift and a low-pass filter (0.1 Hz cutoff) to reduce physiological noise.

### Definition of Seeds for Resting State FC

The sgACC seeds that were used in the FC analysis were based on our previous studies that demonstrated sex differences and sex-specific abnormalities in sgACC FC to regions throughout the brain in healthy individuals and those with chronic pain (3, 18). The sgACC seeds had a radius of 6 mm and were centered around the Montreal Neurological Institute (MNI) coordinates on the right (5, 34, −4) and left (−5, 34, −4). The anatomical location of the sgACC makes this region susceptible to signal dropout. Therefore, we established a minimum cutoff (BOLD signal intensity equal to or exceeding 50% of the mean intensity within all non-zero voxels in the brain) that each seed in each participant must reach for inclusion in the analysis. Two additional spherical seeds were created to examine the FC of key nodes in the descending pain modulation system, the PAG, and RVM. Each seed had a 3 mm radius and was centered around coordinates used previously in our lab (47, 48), MNI: 0, −32, 10 (PAG) and MNI: 0, −34, −50 (RVM).

### Functional Connectivity Analyses

#### Analysis 1: sgACC to Whole Brain FC

In analysis 1, we assessed whether whole brain sgACC FC was abnormal in CTS patients compared with age/sex-matched HCs. To do this, we first took the right and left sgACC seeds and transformed them non-linearly from standard space into native space for each participant. Next, the average time series for each seed was extracted and used in a first-level GLM analysis in FSL's FEAT that calculated the sgACC seed-to-whole brain FC of each seed for every participant (49). In each GLM, the time series of the sgACC seed region was entered as a regressor to identify voxels throughout the whole brain that were significantly functionally connected to the seed. The output from each participant's first-level analysis was then entered into a second-level group analysis, using FEAT's two-group difference adjusted for covariates model and FLAME 1 + 2 (50). This consisted of a two-sample unpaired *t*-test, comparing CTS patients to HCs with two additional covariates included in the analysis: sex and age. Including these covariates in the model allowed us to examine group differences (CTS vs. HC), adjusted for the participants' mean—centered age and sex. Additionally, we included positive and negative contrasts for each covariate. These contrasts revealed where in the brain sgACC FC was greater or reduced for all the participants (CTS and HCs), depending on sex (men/women) or age (younger/older) as a continuous variable. Sex was included because our previous study found sex differences and sex-specific abnormalities in sgACC FC in another chronic pain condition (18). While our current sample had insufficient numbers of men (*n* = 7) to directly compare sex differences among the CTS patients, we, nonetheless, wanted to explore if sex had any effects on sgACC FC in CTS. Statistical significance was determined using Family Wise Error (FWE) correction with a cluster-based threshold of *Z* > 2.3 and *P* < 0.05.

#### Analysis 2: Exploratory Sex-Disaggregated sgACC to Whole Brain FC

To further explore the significant positive effect of sex revealed in Analysis 1, we performed two exploratory sex-disaggregated analyses of whole brain sgACC FC. To do this, first-level sgACC to whole brain GLMs for women with CTS (*n* = 18) and HC women (*n* = 18) were entered into a second-level group analysis with FEAT's two-group difference model as before; this time, with only age included as a covariate. The same process was repeated in a second-level FEAT analysis, comparing men with CTS (*n* = 7) and HC men (*n* = 7). Statistical significance for these sex-disaggregated analyses was determined using FWE correction with a cluster-based threshold of *Z* > 2.3 and *P* < 0.05.

#### Analysis 3: sgACC and the Descending Pain Modulation System

To investigate the FC of the sgACC with the PAG and RVM, we included an automated brain stem co-registration (ABC) (51) as an additional preprocessing step. This step involved linear registration from each participant's anatomical space to standard MNI152 2-mm space as before, followed by a second linear registration step from anatomical to standard space, using a binarized brain stem mask, created with the Harvard Oxford Subcortical Atlas. This was used as a reference volume to weight FLIRT's correlation ratio cost function and allow focussed alignment of the brain stem. Each participant's transformed matrix (from the registration of his or her functional data to anatomical space created during FEAT preprocessing) was concatenated with the matrices from ABC, and then FLIRT was used to create the inverse of this concatenated transformation matrix. The inverse matrix was used to transform the 3-mm radius spherical PAG and RVM seeds (created in standard MNI152 2-mm space) to each participant's functional space, and the average time series were extracted for each seed. First-level seed to whole brain FC analyses were performed (as in Analysis 1) for the PAG and RVM seeds. We combined our right and left sgACC seeds to create an sgACC “mask” and applied this mask to a second-level FEAT analysis (a two-group difference model) to compare PAG-sgACC FC between CTS and HC, with sex and age again included as covariates. We repeated this second-level analysis with the RVM. As before, statistical significance was determined using FWE correction with a cluster-based threshold of *Z* > 2.3 and *P* < 0.05. Finally, we correlated the averaged time series extracted from our PAG and RVM seed, using MATLAB (R2016b) to calculate FC between the PAG and RVM. A Fisher r-to-z transform was performed to compare FC between these two ROIs in CTS patients with HCs (two-sample *t*-test, *p* < 0.05).

#### Analysis 4: Treatment-Related Plasticity in sgACC FC

To examine treatment-related plasticity in sgACC FC, we performed a paired two-group difference (two-sample paired *t*-test) analysis in FEAT, comparing pre- to post-op sgACC to whole brain FC in 17 of our CTS patients (13 women, 4 men). Statistical significance was determined using FWE correction with a cluster-based threshold of *Z* > 2.3 and *P* < 0.05.

## Results

### Descriptive Statistics and Clinical Characteristics of Patients With CTS

**Figure 1:**
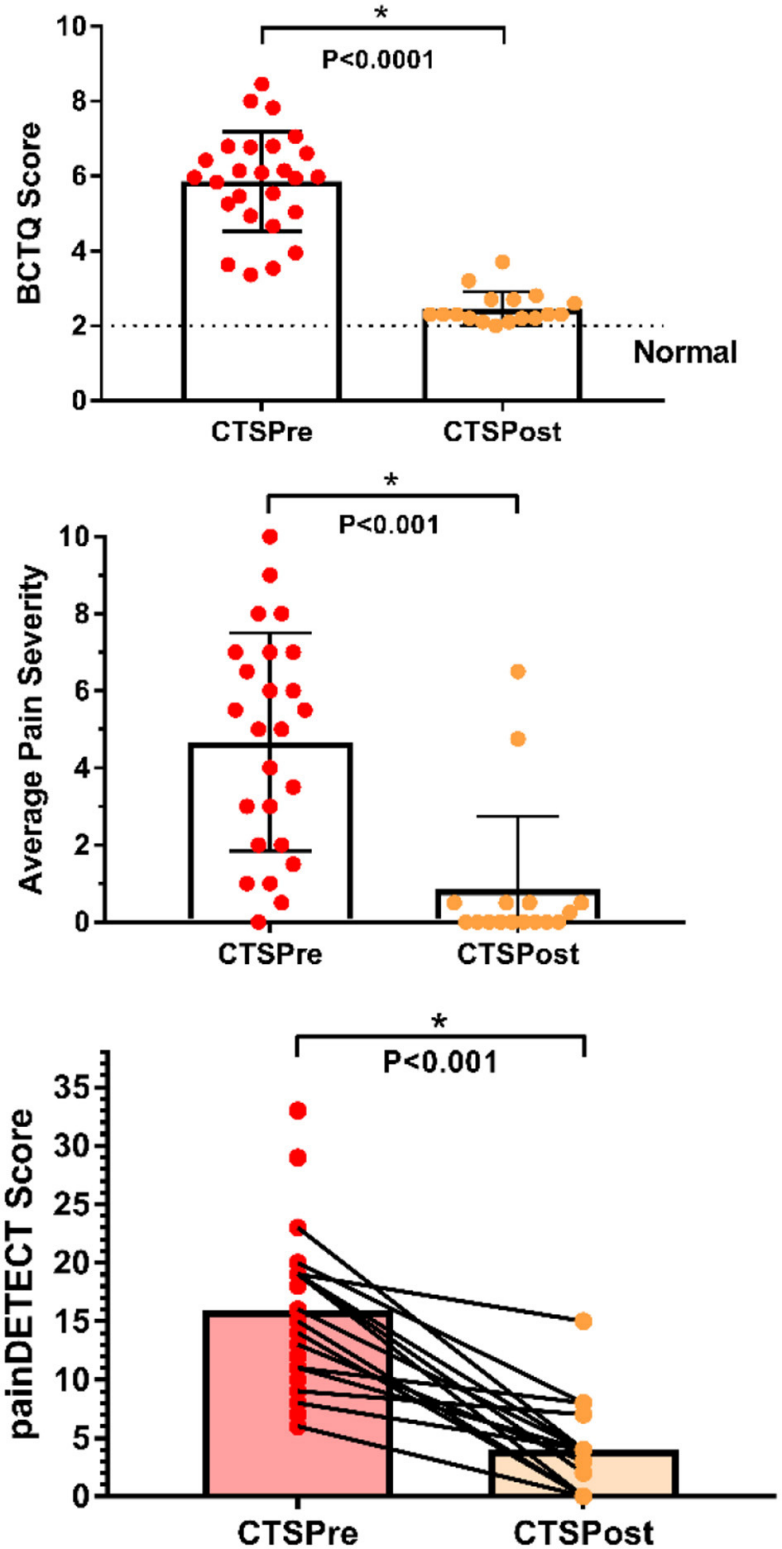
Clinical characteristics of chronic pain patients pre and post carpal tunnel release surgery. CTS symptoms and functional impairment pre-op (*n* = 25) improved significantly with surgical treatment (post-op *n* = 17) as measured by BCTQ scores (2 = normal, 10 = most severe). Average pain severity score was calculated by taking an average of the scores from the Brief Pain Inventory for best and worst pain in the past 24 h, pain at present, and average pain for each CTS patient (0 = no pain, 10 = pain as bad as you can imagine). Average pain severity was significantly reduced following surgical treatment. Neuropathic pain scores (measured by painDETECT) were also significantly reduced following surgery. *P < 0.001 paired *t*-test *n* = 17 patient's pre- and post-op scores; BCTQ, Boston carpal tunnel questionnaire; CTS, carpal tunnel syndrome; HC, healthy control.

Clinical and demographic information for patients and healthy participants are presented in Table 1 and Figure 1. Among the HCs, two-sample *t*-tests showed there were no significant sex differences in age (*P* = 0.72) or depression scores (*P* = 0.57). There were no significant differences in age between patients and HCs, but CTS patients had significantly higher depression scores than HCs (CTS average ± SD, 9.9 ± 7.2, compared with HC average ± SD, 5.2 ± 6.3, two-sample *t*-test *P* = 0.01). Only three of the CTS patients' depression scores were in the “moderate” range, with four classified as “mild” and the majority falling in the “none to minimal” category (see Supplementary Figure 1). Two CTS patients self-reported a diagnosis of depression, while three had a diagnosed anxiety disorder. Our cohort consisted of 22 patients with bilateral CTS symptoms and three with unilateral CTS. There was a predominance of right hands with more severe symptoms (*n* = 16) compared with the left being more severe (*n* = 9). Within the pre-op CTS patient group, two-sample *t*-tests revealed there were no significant sex differences in age (*P* = 0.73), depression (*P* = 0.72), BCTQ score (*P* = 0.81), and average pain (*P* = 0.33). There were also no significant sex differences in Pain DETECT scores (*P* = 0.87). Pre-operatively, nine patients had Pain DETECT scores below 13 (indicating a low likelihood of a neuropathic pain component), six had scores of 13–18 (indicating their pain may have a neuropathic component), and 11 had scores above 19 (indicating a high likelihood of neuropathic pain).

**Table 1:** Demographic and clinical characteristics.

		**CTS Pre-Op**	**CTS Post-Op**	**HCs**
*N*	Total	25	17	25
	W/M	18/7	13/4	18/7
Age (years)	Total	49.6 ± 12.0	52.1 ± 12.0	45.7 ± 9.4
	W/M	W: 49.9 ± 12.2	W: 52.1 ± 12.5	W: 46.3 ± 10.4
		M: 48.4 ± 11.4	M: 50.3 ± 11.8	M: 44.7 ± 8.2
Depression (BDI, 0 to 63)	Total	9.9 ± 7.2*	4.8 ± 6.9**	5.2 ± 6.3*
	W/M	W: 9.5 ± 7.5		W: 4.9 ± 6.7
		M: 10.7 ± 7.0		M: 6.6 ± 5.6
BCTQ (score: 2–10)		5.9 ± 1.3	2.5 ± 0.4**	
painDETECT (score: 0–38)		15.9 ± 6.4	4.0 ± 3.8**	
BPI Average pain severity (score: 1–10)		4.7 ± 2.8	0.8 ± 1.9**	

Group data are displayed as mean ± standard deviation. Scores with significant group differences are indicated with an * for the pre-op CTS vs. HC comparison and ** for the subset of CTS patients with pre vs. post-op comparisons. Beck's depression inventory (BDI) scores were missing from three pre-op carpal tunnel syndrome (CTS) patients, and the average pain severity score was missing from one post-op CTS patient. BCTQ, Boston carpal tunnel questionnaire; BPI, brief pain inventory; HCs, healthy controls; W, women; M, men.

Among the patients who returned for a post-op study visit, eight had surgery on both hands, and nine had surgery only on the most symptomatic hand (6 right, 3 left). Within the patient group, pain DETECT scores fell significantly from 14.9 ± 4.9 pre-op to 4.0 ± 3.8 post-op (*P* < 0.001, paired one-tailed *t*-test). Furthermore, pain ratings significantly dropped by an average of 81.1%, from pre-op levels of 4.3 ± 2.8 to post-op levels of 0.8 ± 1.9 (*P* < 0.001, paired one-tailed *t*-test). Similarly, BCTQ scores improved on average by 3.1 ± 1.0 (pre-op: 5.6 ± 1.1, post-op: 2.5 ± 0.4, *P* < 0.0001), resulting in an average post-op BCTQ score of 2.5 ± 0.5 for the right hand and 2.5 ± 0.6 for the left. Depression scores also improved following surgical treatment (pre-op: 7.5 ± 6.8, post-op 4.8 ± 6.9, *P* = 0.05, paired one-tailed *t*-test).

### Abnormal sgACC FC With Medial Pre-frontal Cortex and Temporal Lobe in Patients With Chronic Pain (Analysis 1)

**Figure 2:**
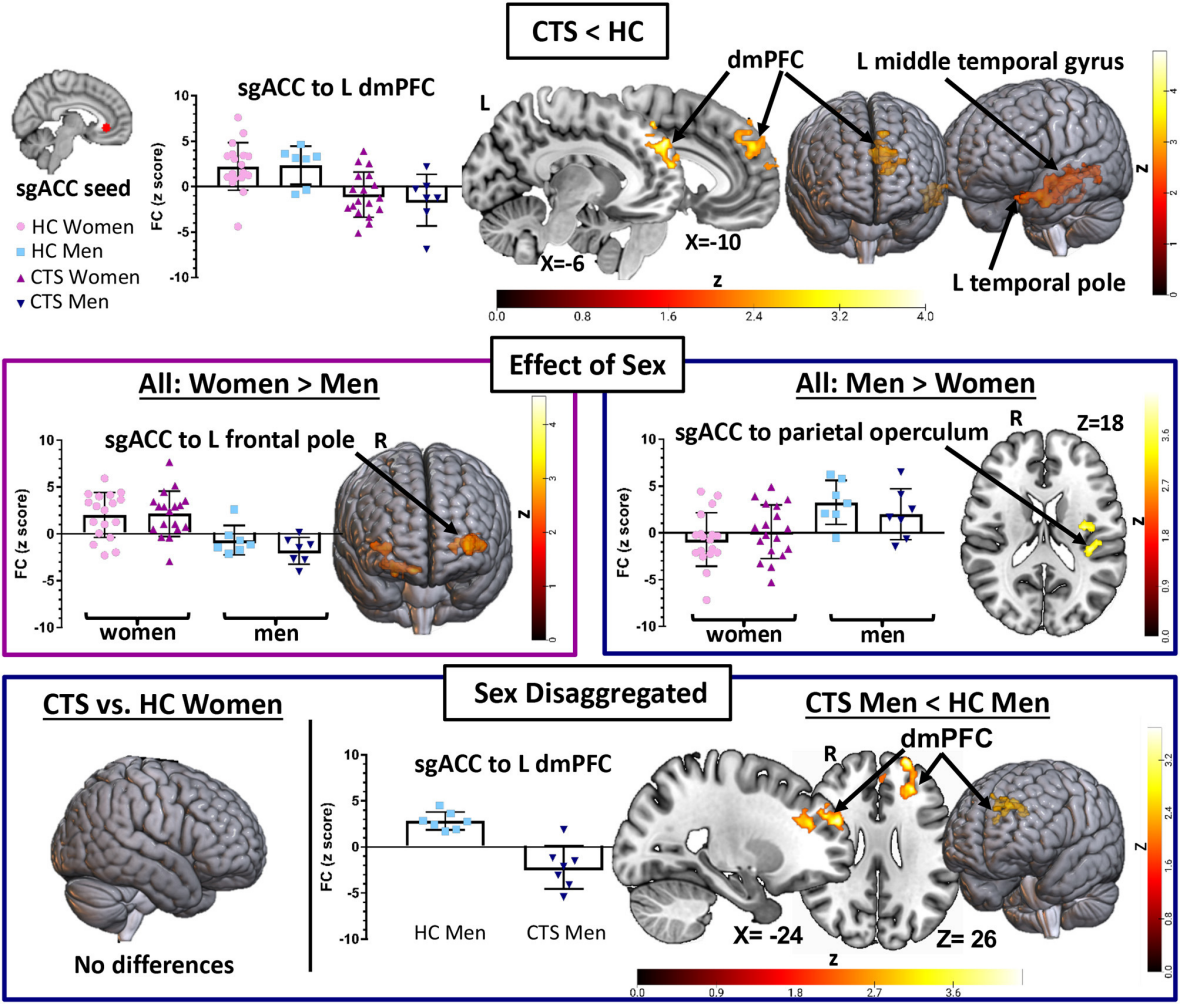
sgACC seed to whole brain functional connectivity analysis reveals abnormalities in pre-op chronic pain patients driven by men. A seed-to-voxel whole brain functional connectivity (FC) analysis compared pre-op CTS patients (*n* = 25) to healthy controls (HCs, *n* = 25) with age and sex included as covariates (Analysis 1). CTS patients had reduced left sgACC FC with the left dmPFC and middle temporal gyrus and reduced right sgACC FC with the left temporal pole when compared with HCs. Significant effects of sex were also found. Bilateral sgACC FC with bilateral frontal poles/ventral pre-frontal cortices was greater in women compared with men, while men had greater sgACC FC with the parietal operculum compared with women. Sex-disaggregated analyses were performed as a follow-up to the significant effect of the sex covariate found in Analysis 1. The sgACC seed-to-voxel whole brain FC analysis, comparing women with CTS (*n* = 18) and healthy women (*n* = 18), did not reveal any significant differences in sgACC FC. However, the analysis comparing men with CTS (*n* = 7) with healthy men (*n* = 7) with age as a covariate showed reduced sgACC FC with the left dmPFC, SMA, and inferior temporal gyrus. Graphs represent the averaged z-scores from the participants in each group (horizontal lines represent the mean, and the vertical lines represent the SD), extracted from a 2-mm spherical seed centered on the peak coordinates of one of the significant clusters from the contrast. Statistical threshold was p <0.05 (FWE-corrected for multiple comparisons). Other significant effects found in Analysis 1 and the men-only analysis are described in Table 2. Significant age effects in Analysis 1 are shown in Figure 3. sgACC, subgenual anterior cingulate cortex; dmPFC, dorsomedial pre-frontal cortex; SMA, supplementary motor area; CTS, carpal tunnel syndrome; W, women; M, men; L, left; R, right; Inf, inferior.

The aim of our first analysis was to determine whether sgACC to whole brain FC was abnormal in people with chronic pain associated with CTS compared with HCs. This analysis showed that the chronic pain group had reduced sgACC FC with several brain regions (see Table 2, Figure 2). Specifically, there was reduced FC of the left sgACC with the left frontal pole/dorsomedial pre-frontal cortex (dmPFC) (*P* < 0.001) and the left middle temporal gyrus (*P* = 0.01) compared with HCs. Also, the right sgACC had reduced FC with the left temporal pole (*P* < 0.001). Because CTS patients had significantly higher depression scores than HCs, and the sgACC is a region that has been linked with major depressive disorder, we correlated patients' BDI scores with a FC metric (i.e., mean zstat extracted from a 2-mm sphere centered around the cluster peak) for the dmPFC, middle temporal gyrus, and temporal pole. There were no significant correlations between BDI scores and sgACC FC with the left medial PFC (Rho = −0.09, *P* = 0.67), left middle temporal gyrus (Rho = −0.23, *P* = 0.30) or left temporal pole (Rho = −0.02, *P* = 0.94; see Supplementary Figure 1).

**Table 2:** Subgenual anterior cingulate cortex (sgACC) functional connectivity in carpal tunnel syndrome chronic pain patients and healthy controls: Main findings.

**Contrast**	**sgACC**	***Z***	***p***	**MNI coordinates**			**Brain region**
				**x**	**y**	**z**	
**Analysis 1. All CTS vs. HC (*****n*** **=** **50)**							
HC > CTS	L	4.19	0.00114	−14	60	18	Left frontal pole, pre-frontal cortex
	L	3.31	0.0133	−54	−26	−12	Left middle temporal gyrus
	R	4.9	1.2e-07	−48	14	−28	Left temporal pole
W > M (effect of sex)	L	4.53	0.0147	−30	66	6	Left frontal pole
	L	4.58	0.0323	26	40	−18	Right frontal pole, orbitofrontal cortex
	R	4.57	0.001	−34	64	0	Left frontal pole
	R	4.17	0.00175	26	42	−16	Right frontal pole, orbitofrontal cortex
M > W (effect of sex)	R	3.33	0.0484	−40	−30	18	Parietal operculum
**Analysis 2. Sex disaggregated, men only (*****n*** **=** **14)**							
HC > CTS	L	4.23	0.016	−26	60	28	Left frontal pole
	R	4.52	7.4e-05	−24	58	26	Left frontal pole
	R	4.57	0.001	−50	−8	−28	Inferior temporal gyrus, middle temporal gyrus
	R	3.7	0.0138	−6	−14	56	Supplementary motor area
	R	3.23	0.0364	−44	8	36	Middle frontal gyrus
**Analysis 4. Pre vs. post-op CTS (*****n*** **=** **17)**							
Pre > Post	L	4.74	1.85e-06	20	20	−14	Right orbitofrontal cortex, right putamen, right caudate
	L	3.45	0.00047	−22	32	−12	Left orbitofrontal cortex, left caudate
	L	3.72	0.036	−38	−64	−58	Left cerebellum
	R	3.83	0.00733	−38	−62	−58	Left cerebellum
	R	4.12	0.0472	44	0	58	Premotor cortex, middle frontal gyrus
Post > Pre	R	3.73	2.8e-06	22	−70	20	Cuneus, precuneus
	R	3.98	0.00484	−36	−16	10	Left posterior insula, central opercular cortex, Heschl's gyrus

*Peak MNI coordinates, Z, and p-values are reported for significant clusters found in the sgACC seed-to-voxel whole brain functional connectivity analyses, comparing carpal tunnel syndrome (CTS) patients (n = 25) and healthy controls (HCs) (n = 25), as well as the sex-disaggregated (n = 14) and CTS pre vs. post-op (n = 17) seed-to-voxel whole brain analyses. Thresholded at p < 0.05 (FWE-corrected for multiple comparisons). W, women; M, men*.

**Figure 3:**
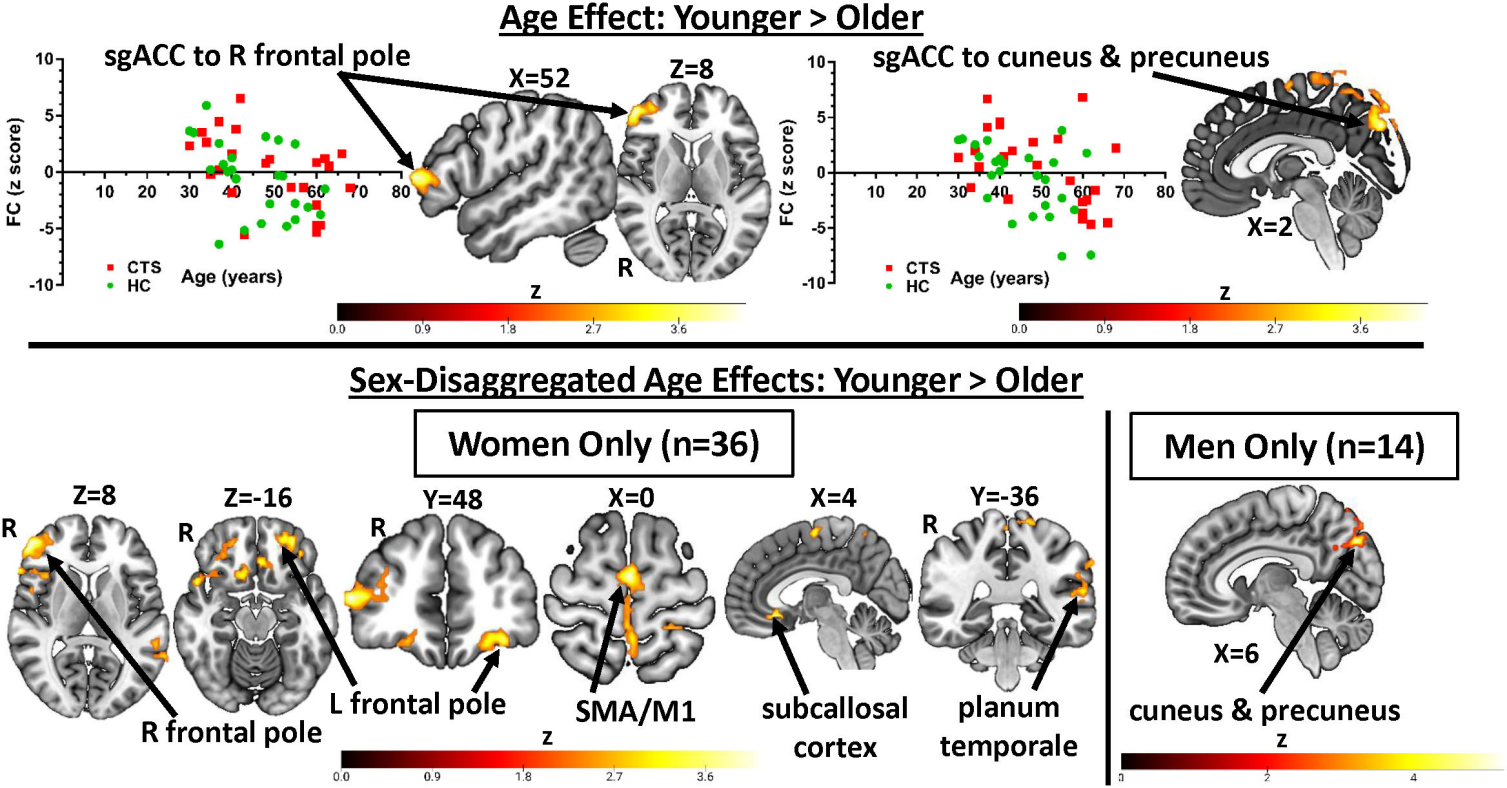
sgACC FC is influenced by age: greater sgACC FC to pre-frontal cortex and precuneus in younger participants. Significant effects of age on sgACC FC (younger > older) found in Analysis 1 (all CTS patients vs. HCs, *n* = 50) as well as the sex-disaggregated analyses (Analysis 2) are presented here. Younger CTS patients and HCs had greater sgACC FC with the right lateral pre-frontal cortex as well as the cuneus and precuneus. The latter finding was also seen in the men-only analysis (*n* = 14), while the women-only analysis (*n* = 36) showed significantly greater sgACC FC with bilateral pre-frontal cortex, orbital frontal cortex, SMA, M1, subcallosal cortex, and planum temporale. Graphs represent the averaged z-scores from the participants in each group (horizontal lines represent the mean, and the vertical lines represent the SD), extracted from a 2-mm spherical seed centered on the peak coordinates of one of the significant clusters from the contrast. Statistical threshold was *p* < 0.05 (FWE-corrected for multiple comparisons). CTS, carpal tunnel syndrome; HCs, healthy controls; R, right; PFC, pre-frontal cortex; SMA, supplementary motor area; M1, primary motor cortex; FC, functional connectivity; sgACC, subgenual anterior cingulate cortex.

In Analysis 1, we also included two covariates to examine the effect of sex and age in the combined group of CTS patients and HCs. We found that women exhibit greater bilateral sgACC FC with bilateral frontal poles and right orbitofrontal cortex compared with men (left sgACC to left frontal pole *P* = 0.01, right sgACC to left frontal pole *P* < 0.001; left sgACC to right frontal pole *P* = 0.03, right sgACC to right frontal pole *P* < 0.01). We also found that men had greater right sgACC FC with the parietal operculum (*P* = 0.04) compared with women (Figure 2, Table 2). There were no areas in the brain where sgACC FC was stronger in the older participants compared with the younger participants. However, we did find several regions where sgACC FC was greater as age decreased, e.g., in younger compared with older participants, including the right frontal pole (left sgACC *P* = 0.03, right sgACC *P* < 0.01), cuneus, and precuneus (*P* < 0.001; see Figure 3; Table 3).

**Table 3:** Negative age effects on subgenual anterior cingulate cortex (sgACC) functional connectivity.

**Contrast**	**sgACC**	***Z***	***p***	**MNI coordinates**			**Brain region**
				**x**	**y**	**z**	
**Analysis 1. All CTS vs. HC analysis (*****n*** **=** **50)**							
Younger > Older	L	4.51	0.0287	52	48	8	Right frontal pole
	R	4.4	2.68e-06	2	−78	36	Cuneus, precuneus
	R	4.24	0.00248	52	48	8	Right frontal pole
**Analysis 2. Sex disaggregated, men only (n** **=** **14)**							
Younger > Older	R	5.44	0.00042	6	−78	36	Cuneus, precuneus
**Analysis 2. Sex disaggregated, women only (*****n*** **=** **36)**							
Younger > Older	R	4.02	2.38e-07	4	26	−12	Subcallosal cortex
	R	4.18	0.00218	0	−12	66	Supplementary motor area, precentral gyrus
	R	4.21	0.00453	52	48	8	Right frontal pole
	R	3.79	0.00938	−32	48	−16	Left frontal pole
	R	3.26	0.0168	−62	−36	16	Planum temporale

*Peak MNI coordinates, Z, and p-values are reported for significant clusters in the negative effect of age contrast, which shows where in the brain there is increasing sgACC functional connectivity with decreasing (younger) age. Significant negative effects of age were found in Analysis 1 (All CTS vs. HC), as well as the sex-disaggregated seed-to-voxel whole brain analyses. Thresholded at p < 0.05 (FWE-corrected for multiple comparisons). W, women; M, men*.

### Men With CTS Have Reduced sgACC FC With the Medial Pre-Frontal Cortex (Analysis 2)

We next ran exploratory sex-disaggregated analyses to examine within-sex FC in the HCs and patients with chronic pain (Figure 2, Table 2). In the women-only analysis, we did not find any significant differences in sgACC to whole brain FC between healthy women (*n* = 18) and women with CTS (*n* = 18), using an FWE correction with a cluster-based threshold of *Z* > 2.3 and *P* < 0.05. However, the men-only analysis (Table 2, Figure 2) revealed that men with CTS (*n* = 7) had reduced bilateral sgACC FC to the left frontal pole and dmPFC (left sgACC *P* = 0.016, right sgACC *P* < 0.001) compared with HC men (*n* = 7). The men with CTS also had decreased right sgACC FC to the inferior and middle temporal gyrus (*P* < 0.001), supplementary motor area (*P* = 0.01), and middle frontal gyrus (*P* = 0.04) compared with HC men.

Sex-specific age effects can be found in Table 3 and Figure 3. In the men-only analysis, younger men had greater right sgACC FC to the cuneus and precuneus (*p* < 0.001) than older men. The women-only analysis revealed that younger women had greater right sgACC FC to the subcallosal cortex (*P* < 0.001), supplementary motor area (*P* = 0.002), right (*P* < 0.001) and left (*P* < 0.01) frontal pole and planum temporal (*P* = 0.02).

### No Chronic Pain-Related Abnormalities in Brain Stem Functional Connectivity With the sgACC (Analysis 3)

Our next analysis focused on the FC of the sgACC with the PAG and RVM. In this analysis, there were no significant differences between the CTS patients and HCs in bilateral sgACC FC with the PAG or the RVM, or between the PAG and RVM (FWE corrected).

### Treatment-Related Plasticity in sgACC FC (Analysis 4)

**Figure 4:**
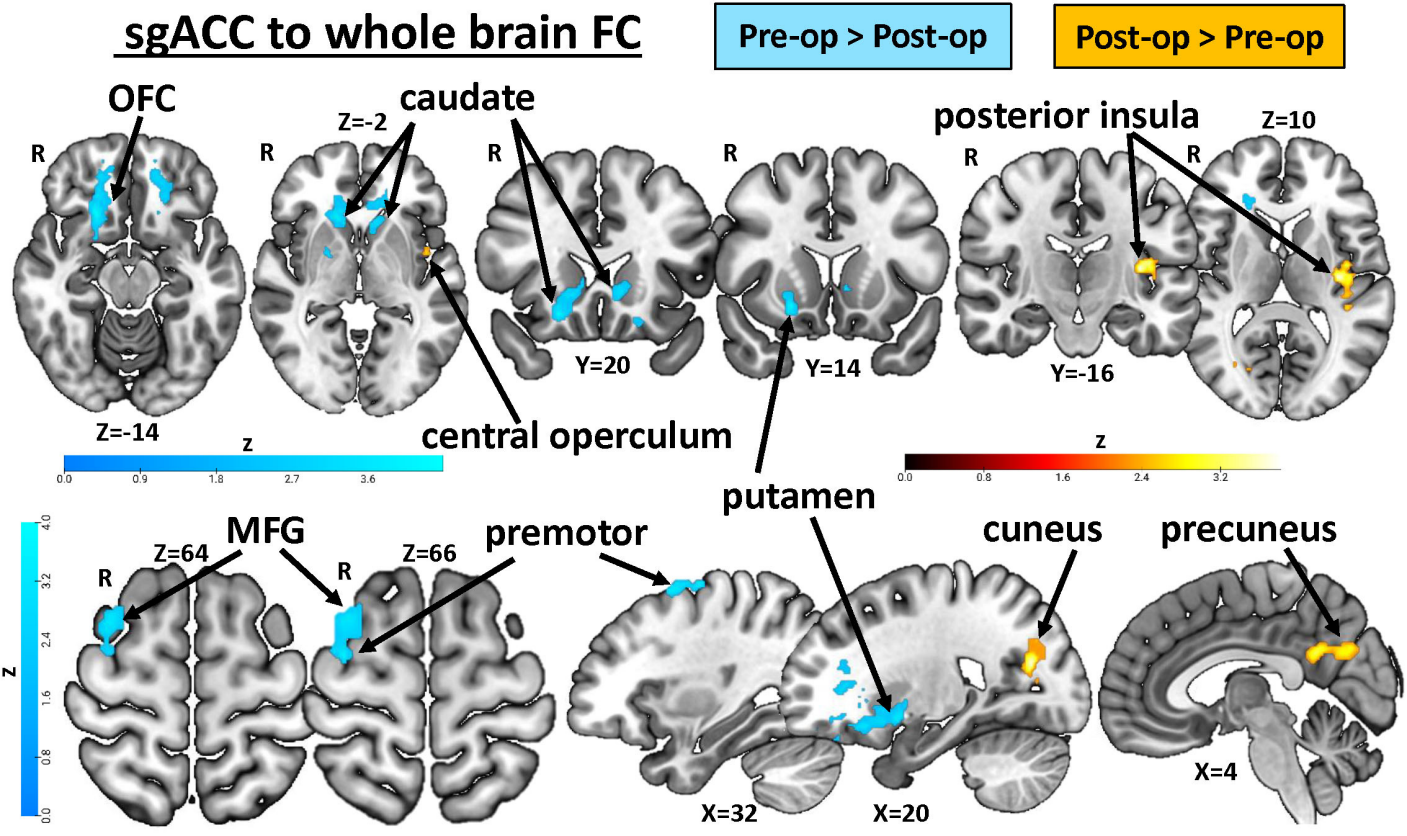
Treatment-related plasticity in sgACC FC in chronic pain patients. A paired two-group difference analysis of CTS patients' pre- and post-op sgACC seed-to-voxel whole brain FC (n = 17) revealed significant differences following surgical treatment. Compared with pretreatment levels, sgACC FC with the orbitofrontal cortex, putamen, caudate, premotor cortex, and middle frontal gyrus was reduced, while FC with the cuneus, precuneus, posterior insula, and central and parietal operculum increased after treatment. Images are thresholded at *p* < 0.05, FWE-corrected for multiple comparisons. R, right; OFC, orbital frontal cortex; MFG, middle frontal gyrus; FC, functional connectivity; CTS, carpal tunnel syndrome; sgACC, subgenual anterior cingulate cortex.

In our last analysis, we examined the impact of carpel tunnel surgery on the FC of the sgACC (see Figure 4, Table 2). We found that following surgical treatment of their CTS, patients (*n* = 17) showed reduced left sgACC FC with the right orbitofrontal cortex, putamen, and caudate (*P* < 0.0001) and left orbitofrontal cortex and caudate (*P* < 0.001) compared with their pretreatment FC. There was also reduced post-op FC between the right sgACC and the right premotor cortex and middle frontal gyrus (*P* = 0.047) as well as bilateral sgACC FC to the left cerebellum (left sgACC, *P* = 0.036: right sgACC, *P* = 0.007) compared with pre-op levels. Conversely, right sgACC FC with the right cuneus and precuneus (*P* < 0.0001) and left posterior insula and central and parietal opercular cortex (*P* < 0.01) increased following surgical treatment. Using the mean zstat extracted from a 2-mm sphere centered around the cluster peak as our FC metric, we investigated whether post-op sgACC functional connectivity to the posterior insula and precuneus/cuneus was correlated with the changes in the patients' BCTQ scores. We found no significant correlations between improvements in BCTQ scores and sgACC FC with the cuneus (Rho = 0.23, *P* = 0.36), precuneus (Rho = 0.32, *P* = 0.21) or the posterior insula (Rho = 0.32, *P* = 0.22; see Supplementary Figure 2).

## Discussion

**Figure 5:**
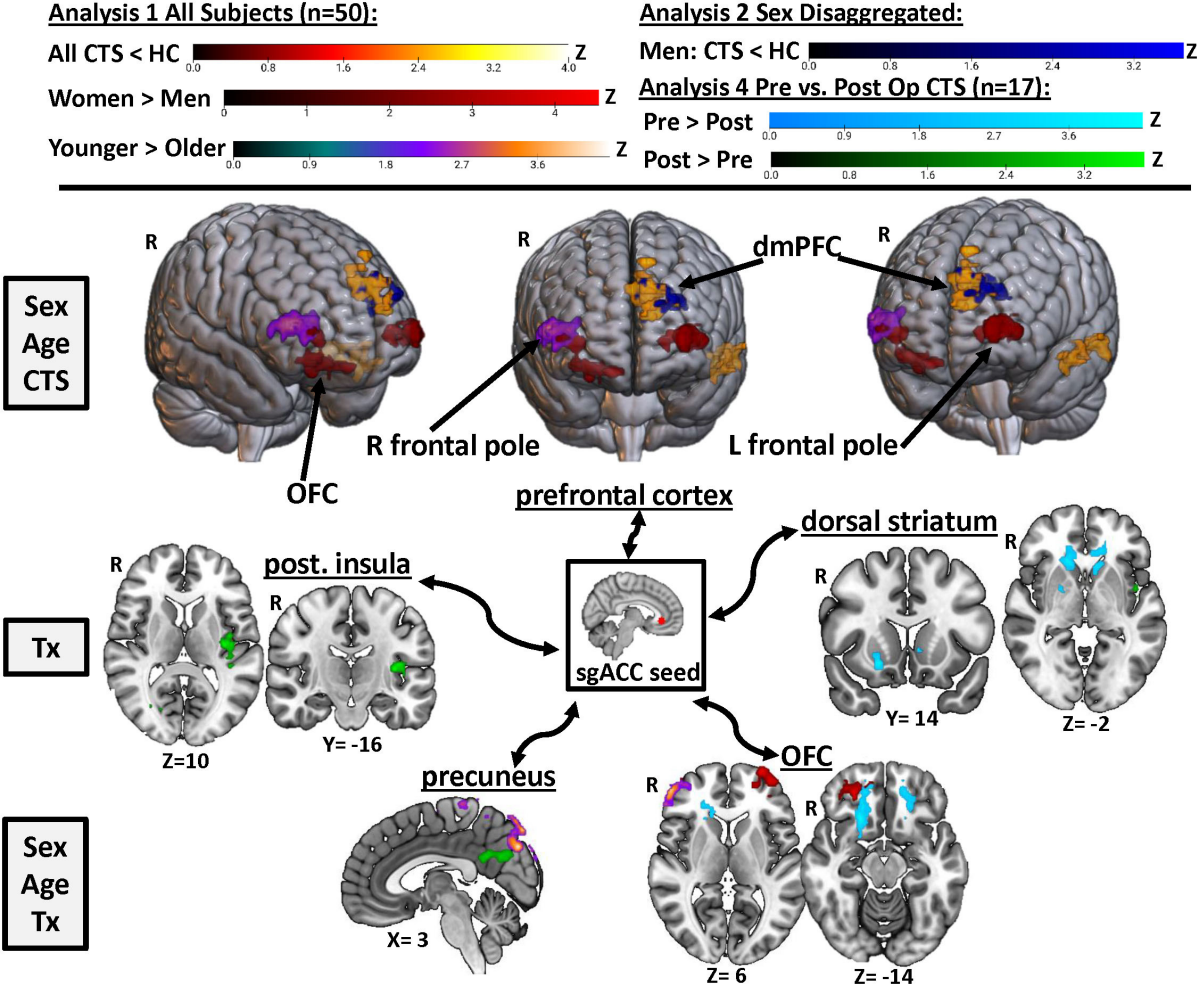
Summary of the effects of chronic pain, treatment, sex, and age on sgACC FC. Results from Analyses 1, 2, and 4 are displayed here to visualize key regions in the brain where sgACC functional connectivity was affected by chronic pain (yellow/orange and royal blue), sex (red), age (purple), and surgical treatment (light blue and green). Images are thresholded at *p* < 0.05, FWE-corrected for multiple comparisons. R, right; Post., posterior; OFC, orbitofrontal cortex; dmPFC, dorsomedial pre-frontal cortex; Tx, treatment; sgACC, subgenual anterior cingulate cortex; CTS, carpal tunnel syndrome.

This study is the first to determine whether the resting state FC of a key node in the descending pain modulation system, namely, the sgACC, is impacted by chronic pain and surgical treatment to alleviate pain, as well as sex. Our study was conducted in a group of patients with chronic pain caused by the common neuropathy, CTS. Our main findings are summarized in Figure 5. We found that (1) sgACC FC with the mPFC and temporal lobe is lower in CTS than in HCs, (2) this abnormal sgACC FC is specific to men with CTS but not found in women with CTS, compared with healthy controls, (3) there are sex differences in sgACC FC in the chronic pain patients and healthy controls, such that women have greater FC with bilateral frontal poles and men have greater FC with the parietal operculum, and (4) some brain regions show increased sgACC FC in younger vs. older adults, and these age effects differ in women and men. Furthermore, a preliminary exploration of surgical treatment effects found that, compared with pre-op levels, FC of the sgACC is increased with the precuneus and posterior insula and decreased with the OFC and dorsal striatum. These results suggest that FC of the sgACC can exhibit plasticity in response to pain treatment; specifically, that sgACC FC is increased with brain regions implicated in pain intensity and decreased with brain regions associated with negative pain affect when an individual is in a pain-free versus a chronic pain state. Overall, our findings highlight connectivity and sex specificity of the sgACC, a key node of the brain's descending pain modulation system, in a chronic pain condition that shows sex specificity. This provides important insight into understanding mechanisms of chronic pain and potential treatment efficacy in men and women.

### Abnormal FC Between the sgACC and PFC in Chronic Pain

Our findings raise the question of whether sgACC dysfunction is a common feature in the subset of individuals who develop a chronic pain condition that is more prevalent in the other sex. Support for this concept comes from our previous study where we reported that people with a male-prevalent chronic pain condition (e.g., ankylosing spondylitis) exhibit lower sgACC FC with the DLPFC (18). Strikingly, this previous finding was specific to women in a male-prevalent pain condition, while men with AS showed no abnormalities in sgACC FC compared with HCs. In the present study, a sex-disaggregated analysis did not identify any differences in sgACC FC in women with CTS to healthy women. This suggests that abnormal sgACC FC may not be specific to women across pain conditions, as it was the men with the female-prevalent pain condition (CTS) who had abnormal sgACC FC.

In chronic pain, abnormal sgACC-mPFC FC could interfere with the ability to use cognitive strategies to engage the descending pain modulation system or enhance the negative affective component of pain. The mPFC provides the main source of cortical input to the PAG (4, 52–54) and is thought to play an integral role in descending pain modulation (55, 56) and placebo analgesia along with the sgACC (57, 58). Disruption of this endogenous antinociceptive circuit has been shown in rodent models of neuropathic pain (59, 60). In humans, abnormalities in PFC gray matter (61, 62) and function (63) have been reported in multiple chronic pain conditions and may represent a predisposing risk factor for developing chronic pain (64). Due to its role in cognitive and affective pain processing, the medial and dorsolateral PFC is the focus of brain stimulation treatments and psychological interventions designed to modulate cognitive appraisals of pain and decrease negative pain affect (55). Interestingly, FC between the sgACC and PFC predicts response to non-invasive brain stimulation treatment in individuals with major depressive disorder (30–32). Importantly, the reduced sgACC FC to the dmPFC seen in pre-op CTS patients did not correlate with their BDI scores, indicating that these sgACC abnormalities are likely related to the presence of chronic pain rather than elevated depression scores in these patients.

### A Triple Threat: The sgACC's Role in Pain, Depression, and Systemic Inflammation

Research on chronic pain, depression, and peripheral inflammation provides converging evidence that the sgACC plays an important role in all three. The sgACC has been linked with pain affect (65, 66) and pain modulation (7, 8), and abnormal sgACC gray matter and function are key features of mood disorders, including major depressive disorder (67–69). Activity in the sgACC has also been linked with peripheral inflammation (70), a symptom that is shared by both AS (inflamed joints) (71, 72) and CTS (inflamed nerve lining) (23, 73, 74). Increases in peripheral inflammatory markers are positively correlated with sgACC activity and FC at rest and during emotional processing tasks (75–77) as well as resting μ-opioid receptor binding potential and sadness-evoked μ-opioid system activation in the sgACC (78). Exactly how this neuroimmune interplay occurs in the sgACC is not clear, although a recent primate study has found that over-activation of the sgACC reduced vagal tone (79), and low vagal tone is associated with peripheral inflammation and depression (80).

It may be that individual differences in the sensitivity or efficacy of the sgACC response to peripheral inflammation contribute to the development of chronic pain. Alternately, changes in this central modulatory mechanism driven by chronic inflammation could explain the observed abnormal sgACC FC. Systemic inflammatory markers and altered T cell responses correlate to pain and disease severity in some patients with AS (81, 82) and CTS (74), but the efficacy of treatments designed to reduce inflammation may be influenced by factors such as sex (83) and the presence of neuropathic pain and maladaptive brain plasticity (72). A recent imaging study in chronic pain has found increased depression scores were associated with elevated neuroinflammation in the anterior middle cingulate cortex and perigenual ACC and FC of the pgACC with the dorsolateral PFC (84). Future studies are needed to specifically examine how pain symptoms, negative affect, and markers of inflammation correspond to abnormalities in the sgACC, and whether restoring its function could relieve pain.

### FC of the sgACC Shows Sex Differences and Sex-Specific Age Effects

This study builds on previous work in our lab that identified sex differences in sgACC FC in both healthy adults and individuals with chronic pain (3, 9, 17, 18). We found that women had increased sgACC FC with bilateral ventral PFC compared with men, while men had increased sgACC FC with the left parietal operculum. We previously reported greater sgACC FC with the right central and parietal operculum in a large cohort of healthy men compared with healthy women (18). Age also affected sgACC FC, with the younger participants showing greater sgACC FC with the right frontal pole, cuneus, and precuneus compared with the older participants. Sex disaggregating the results revealed these age effects were sex specific, with the former finding driven by women and the latter by men. General-increased FC in younger participants (85) and sex differences in the effects of aging on brain volume (86) and resting state FC (87) are seen in healthy adults. This indicates that it may be important to consider both sex and age in the options for personalized pain treatments that target the sgACC.

### Treatment-Related Plasticity in sgACC FC With Regions Involved in Processing Pain Intensity and Affect

Our preliminary exploration of treatment-related plasticity found that, before surgery, patients had stronger sgACC FC with the orbitofrontal cortex (OFC), dorsal striatum (caudate and putamen), premotor cortex, and middle frontal gyrus compared with after surgery. Striatal dopamine has been associated with pain processing, which is altered in chronic pain (88, 89), and a recently discovered somatosensory-striatal pathway in mice has been linked with comorbid anxiety behaviors in a persistent inflammatory pain model (90). Reduced striatal function has also been linked to depression, anhedonia, and reduced motivation in a reward task in chronic pain patients (91). In the current study, depression scores in our CTS patients were higher pre-treatment compared with post. Future studies would be needed to further examine whether increased sgACC-striatal FC in individuals in chronic pain before treatment correlates with depression or anxiety scores. The OFC is another brain region that had greater pre-op than post-op sgACC FC. The OFC is associated with a variety of cognitive functions and can also exhibit abnormalities in chronic pain. For example, OFC gray matter thickness was positively correlated with neuroticism and negatively correlated with pain unpleasantness ratings in TMD patients (92). In the present study, the OFC regions with increased sgACC FC in pre- vs. post-op CTS patients overlapped with the regions that showed greater FC in younger women.

Post-op, CTS patients had greater sgACC FC with the posterior insula and central/parietal operculum compared with their pre-op scans. Increased sgACC FC with these two regions was previously seen in healthy men compared with women (3, 18). The posterior insula is an important node in the ascending pain pathway responsible for processing the intensity of somatosensory stimuli, including pain (66, 93, 94). Acupuncture treatment-related increases in FC between the left insula and right frontoparietal network are associated with improved pain scores in osteoarthritis (95). Post-op CTS patients also had greater sgACC FC with the cuneus and precuneus compared with their pre-op scans. The precuneus is a key node in the default mode network, and abnormalities in precuneus FC have been associated with rumination and excessive internal monitoring in chronic pain (96, 97). Our previous study found increased sgACC-precuneus FC in healthy women compared with men and increased sgACC-precuneus FC in women with chronic pain compared with healthy women (18). However, in CTS, increased FC between the descending pain modulation system and brain regions associated with self-referential thinking (precuneus/posterior cingulate cortex) is associated with pain-free post-op patients compared with their pre-op state. Acupuncture treatment-related increases in FC between the precuneus and the rostral ACC/mPFC have also been shown in migraine patients (98). Surprisingly, post-op sgACC FC increases with the posterior insula and cuneus/precuneus did not correlate with improved BCTQ scores in our patients. Therefore, plasticity in these regions may be associated with other cognitive or physical aspects of recovery.

### Study Limitations

Our study has several limitations to consider. First, there are relatively low numbers of men with CTS in general, which was reflected in our study sample. Therefore, we were not able to directly examine sex differences in sgACC FC within patients with chronic neuropathic pain from CTS. Despite considerable sex differences in pain biology, prevalence, and treatment response, few studies have conducted sex difference or sex-specific analyses (20). Thus, we importantly conducted sex-disaggregated analyses, especially because sex is known to influence sgACC FC in healthy and chronic pain states (3, 9, 16 −18). However, our men-only analysis had a small sample size, and, therefore, any conclusions drawn from it are limited and best suited to identifying ideas to explore more thoroughly in future, adequately powered investigations. Second, our post-op sample is also relatively small, therefore, a similar caveat must be applied to our comparison of pre- vs. post-op patients, although the within-subject longitudinal design of this analysis is somewhat more robust to smaller sample sizes. Future studies should investigate sex differences in sgACC FC response to treatment and, specifically, whether FC with the mPFC increases once CTS symptoms resolve following treatment, as has been reported with acupuncture treatment of osteoarthritis (99). Another important consideration is whether lateralization in findings (such as those reported here, i.e., reduced sgACC FC with the left dmPFC in pre-op CTS patients vs. HCs, increased post-op sgACC FC with the left posterior insula) is influenced by laterality in CTS symptoms. In our cohort, the right hand was typically more symptomatic and more often operated on than the left, although most patients (*n* = 22) had bilateral CTS. It is unlikely that persistent symptoms in untreated left hands contributed to the left posterior insula findings as average post-op BCTQ scores for both right and left hands were low. A larger study with equally distributed right- and left-dominant CTS could explore how laterality in symptoms and pain-relief influence observed brain changes.

### Future Directions: The sgACC as a Therapeutic Target for Pain Relief

Identifying sex-specific brain alterations could help explain differences in symptom presentation and treatment response and provide clues for sex divergent disease mechanisms in the CNS. Characterizing the effects of sex and age on brain circuitry is important in the development of personalized pain treatments because therapies developed with research based primarily on one sex can be less effective in the other sex (20). Findings from this study in CTS and our previous study in ankylosing spondylitis suggest that dysfunction in the sgACC is present in some individuals with chronic pain, specifically those with conditions that are more prevalent in the opposite sex. Deep brain stimulation of different parts of the ACC has been shown to provide significant, long-lasting pain relief in some chronic pain patients (100, 101). A preliminary trial targeting the sgACC with transcranial direct current stimulation to reduce chronic cluster headaches has shown promising results (102). Therefore, with its role in the descending pain modulation system as well as mood and affective processing, the sgACC could represent a potential target for brain stimulation treatments for chronic pain.

## Data Availability Statement

The datasets presented in this article are not readily available because sharing of any MRI data would be subject to our institutional approval of anonymization. Requests to access the datasets should be directed to karen.davis@uhnresearch.ca.

## Ethics Statement

The studies involving human participants were reviewed and approved by University Health Network Research Ethics Board. The patients/participants provided their written informed consent to participate in this study.

## Author Contributions

NO, KD, and DA conceived the study. DA assisted in patient recruitment for the study, and NO collected the data with assistance from JK, CF, KH, JC, AR, RB, and RE. All authors contributed to the drafting of the manuscript and approved the submitted version.

## Conflict of Interest

The authors declare that the research was conducted in the absence of any commercial or financial relationships that could be construed as a potential conflict of interest.

## Supplementary Material

Supplementary Figure 1 Increased depression scores in chronic pain patients do not correlate with sgACC FC abnormalities. Beck's depression inventory (BDI) scores for pre-op (*n* = 25, data missing from three patients) and post-op (*n* = 17) CTS patients and healthy controls (HCs, *n* = 25). CTS patients' pre-op depression scores were significantly higher than HCs (*P* = 0.01, two-sample *t*-test). Patients' depression scores fell significantly after treatment (*P* = 0.05, *n* = 17, paired *t*-test). Because pre-op patients had increased depression scores and sgACC abnormalities are frequently reported in depression, we correlated patients' pre-op BDI scores with a functional connectivity (FC) metric (mean zstat extracted from a 2-mm sphere centered around the cluster peak) for the brain regions that showed abnormal sgACC FC in patients. There were no significant correlations between patients' pre-op BDI scores and their sgACC FC with the left dorsomedial PFC (dmPFC: Rho = −0.09, *P* = 0.67), left middle temporal gyrus (MTG: Rho = −0.23, *P* = 0.30) or left temporal pole (TP: Rho = −0.02, *P* = 0.94). L, left; sgACC, subgenual anterior cingulate cortex.

Supplementary Figure 2 Change in BCTQ scores do not correlate with post-op increases in sgACC functional connectivity (FC). We investigated whether post-op increases in sgACC functional connectivity to the insula and precuneus/cuneus were associated with the changes in patients' BCTQ scores, using Spearmen's correlation. Graphs show the mean zstat extracted from a 2-mm sphere centered around the cluster peak, correlated with change in a BCTQ score for each post-op CTS patient (*n* = 17). We found no significant correlations between improvements in a BCTQ score and sgACC FC with the cuneus (Rho = 0.23, *P* = 0.36), precuneus (Rho = 0.32, *P* = 0.21) or the insula (Rho = 0.32, *P* = 0.22). BCTQ, Boston carpal tunnel questionnaire; sgACC, subgenual anterior cingulate cortex; R, right.
