# Supplementary figures and images for: Sex-Specific Abnormalities and Treatment-Related Plasticity of Subgenual Anterior Cingulate Cortex Functional Connectivity in Chronic Pain
Source: Front Pain Res (Lausanne). 2021 Jul 12;2:673538. doi: 10.3389/fpain.2021.673538 (PMC8915549; doi:10.3389/fpain.2021.673538)

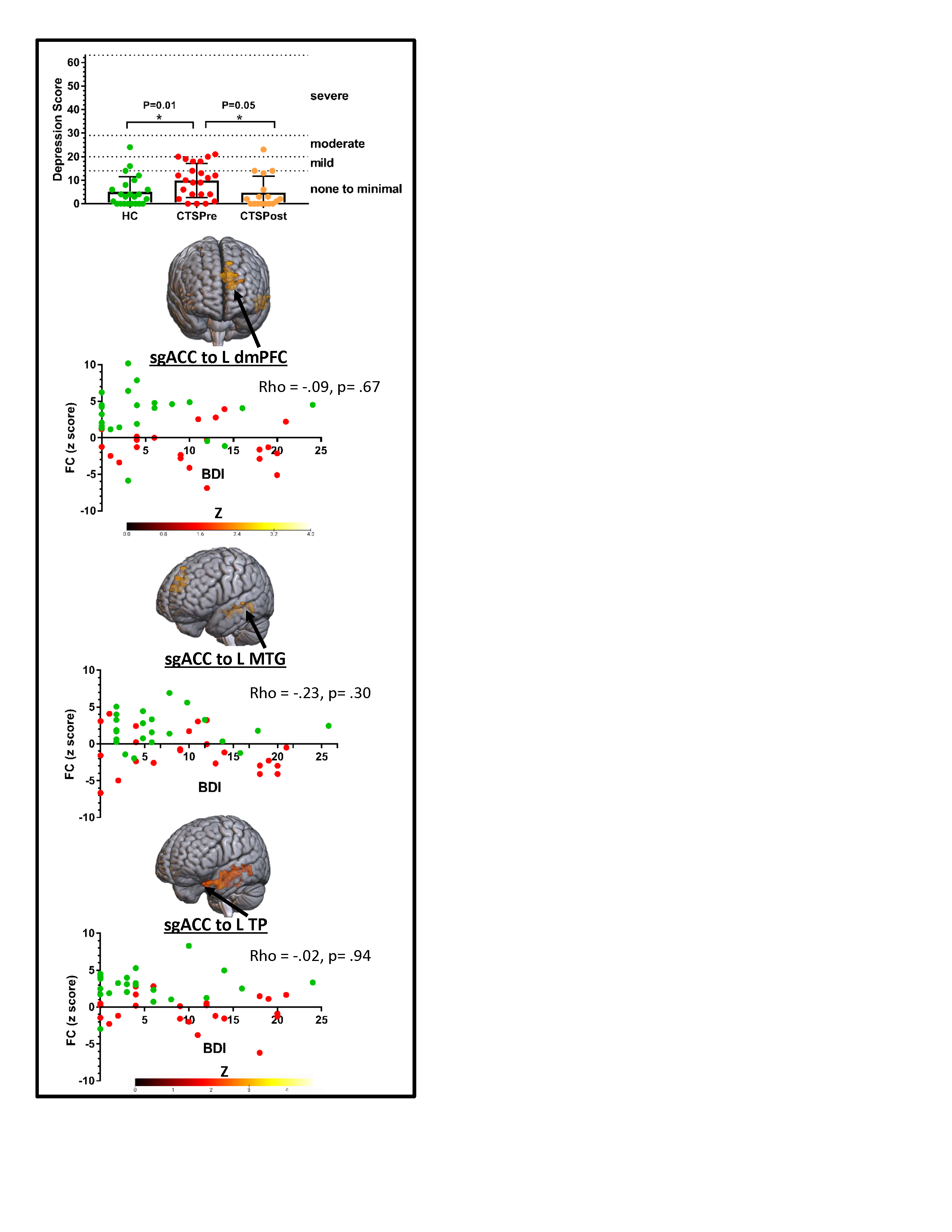

Supplement: Supplementary Figure 1 — Increased depression scores in chronic pain patients do not correlate with sgACC FC abnormalities. Beck's depression inventory (BDI) scores for pre-op (n = 25, data missing from three patients) and post-op (n = 17) CTS patients and healthy controls (HCs, n = 25). CTS patients' pre-op depression scores were significantly higher than HCs (P = 0.01, two-sample t-test). Patients' depression scores fell significantly after treatment (P = 0.05, n = 17, paired t-test). Because pre-op patients had increased depression scores and sgACC abnormalities are frequently reported in depression, we correlated patients' pre-op BDI scores with a functional connectivity (FC) metric (mean zstat extracted from a 2-mm sphere centered around the cluster peak) for the brain regions that showed abnormal sgACC FC in patients. There were no significant correlations between patients' pre-op BDI scores and their sgACC FC with the left dorsomedial PFC (dmPFC: Rho = −0.09, P = 0.67), left middle temporal gyrus (MTG: Rho = −0.23, P = 0.30) or left temporal pole (TP: Rho = −0.02, P = 0.94). L, left; sgACC, subgenual anterior cingulate cortex. [file Image_1.tiff]

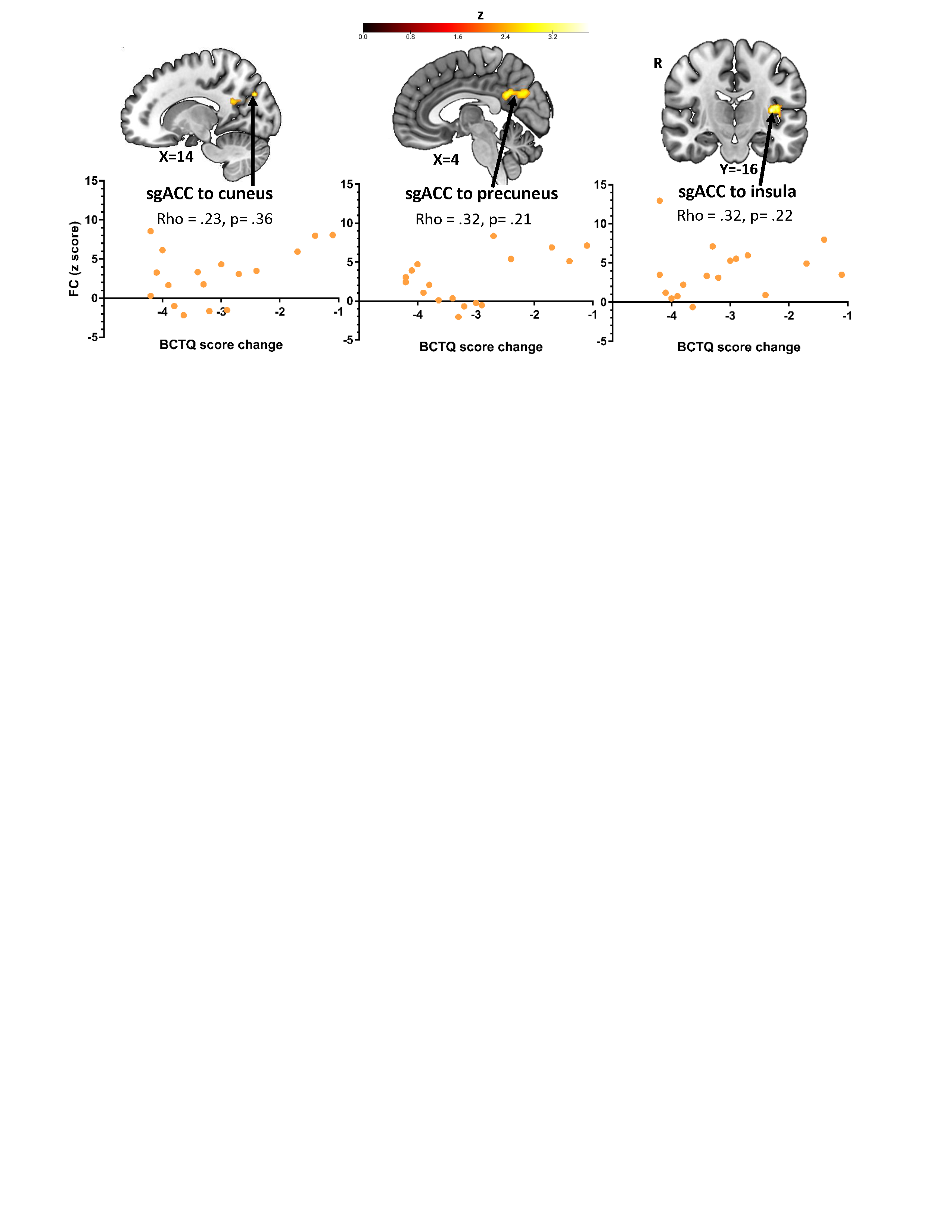

Supplement: Supplementary Figure 2 — Change in BCTQ scores do not correlate with post-op increases in sgACC functional connectivity (FC). We investigated whether post-op increases in sgACC functional connectivity to the insula and precuneus/cuneus were associated with the changes in patients' BCTQ scores, using Spearmen's correlation. Graphs show the mean zstat extracted from a 2-mm sphere centered around the cluster peak, correlated with change in a BCTQ score for each post-op CTS patient (n = 17). We found no significant correlations between improvements in a BCTQ score and sgACC FC with the cuneus (Rho = 0.23, P = 0.36), precuneus (Rho = 0.32, P = 0.21) or the insula (Rho = 0.32, P = 0.22). BCTQ, Boston carpal tunnel questionnaire; sgACC, subgenual anterior cingulate cortex; R, right. [file Image_2.tiff]
